# Predicting ward transfer mortality with machine learning

**DOI:** 10.3389/frai.2023.1191320

**Published:** 2023-08-02

**Authors:** Jose L. Lezama, Gil Alterovitz, Colleen E. Jakey, Ana L. Kraus, Michael J. Kim, Andrew A. Borkowski

**Affiliations:** ^1^James A. Haley Veterans' Hospital, United States Department of Veterans Affairs, Tampa, FL, United States; ^2^Division of General Internal Medicine, Department of Internal Medicine, Morsani College of Medicine, USF Health, Tampa, FL, United States; ^3^National Artificial Intelligence Institute, Washington, DC, United States; ^4^Department of Surgery, Morsani College of Medicine, USF Health, Tampa, FL, United States; ^5^Department of Pathology and Cell Biology, Morsani College of Medicine, USF Health, Tampa, FL, United States

**Keywords:** intensive care units, ward transfer, machine learning, predictive medicine, medical analytics, AI, medicine, surgery

## Abstract

In order to address a long standing challenge for internal medicine physicians we developed artificial intelligence (AI) models to identify patients at risk of increased mortality. After querying 2,425 records of patients transferred from non-intensive care units to intensive care units from the Veteran Affairs Corporate Data Warehouse (CDW), we created two datasets. The former used 22 independent variables that included “Length of Hospital Stay” and “Days to Intensive Care Transfer,” and the latter lacked these two variables. Since these two variables are unknown at the time of admission, the second set is more clinically relevant. We trained 16 machine learning models using both datasets. The best-performing models were fine-tuned and evaluated. The LightGBM model achieved the best results for both datasets. The model trained with 22 variables achieved a Receiver Operating Characteristics Curve-Area Under the Curve (ROC-AUC) of 0.89 and an accuracy of 0.72, with a sensitivity of 0.97 and a specificity of 0.68. The model trained with 20 variables achieved a ROC-AUC of 0.86 and an accuracy of 0.71, with a sensitivity of 0.94 and a specificity of 0.67. The top features for the former model included “Total length of Stay,” “Admit to ICU Transfer Days,” and “Lymphocyte Next Lab Value.” For the latter model, the top features included “Lymphocyte First Lab Value,” “Hemoglobin First Lab Value,” and “Hemoglobin Next Lab Value.” Our clinically relevant predictive mortality model can assist providers in optimizing resource utilization when managing large caseloads, particularly during shift changes.

## 1. Introduction

Predicting mortality and morbidity amongst hospitalized patients has long been a struggle for inpatient Internal Medicine physicians. Sepsis, in particular, leads to over half the mortality in US Hospitals (Sweeney et al., 2018). Very few recent models offer some hope for an early warning system to predict worsening outcomes from sepsis (Adams et al., 2022; Mayampurath et al., 2022; Nestor et al., 2022; Yan et al., 2022). To prevent physicians from burnout, more hospital organizations are turning to shift work for the care of hospitalized patients, similar to the Internal Medicine training programs that follow the 80-h work limit for physicians set by the Accreditation Council for Graduate Medical Education (ACGME). Such shift work frequently leads to a handoff of patients, with many physicians often being the sole provider in the hospital for close to 100 patients. Handoff tools such as I-PASS and other tools can prove ineffective due to subjectivity, inconsistency of updates, and copying and pasting practices, which can negatively impact care. We propose developing an artificial intelligence model that could predict which patients will be most at risk of increased mortality as a resource for physicians to prioritize which patients they should concentrate on during their shift. Unfortunately, physicians on shift work tend to be reactive to calls of distress from the nursing or other support staff rather than proactive in addressing urgent issues. This can often result in patients requiring transfers to the intensive care unit (ICU). A measure called ward transfer mortality in the Veterans Healthcare System looks at the death rate of patients within 30 days of being transferred to the ICU. This measure is critical in patients with aggressive infections such as sepsis, pneumonia, bacteremia, abscesses, and endocarditis. All these require timely interventions for improved outcomes.

In our model, we attempted to look at patients who were transferred to the ICU at the James A. Haley Veterans' Hospital. Our high complexity facility follows the usual academic center model of having trainees such as residents and fellows working with attending staff. We examined patients with significant infectious states and those with pulmonary, neurological, and cardiology diagnoses. In addition, we looked at the following factors concerning their ward transfer mortality:

- Drop in Leukocyte count of 50 percent or greater before they were deemed to require transfer to the ICU;
- Drop in Hemoglobin level of 25 percent or greater before they were deemed to need transfer to the ICU;
- Presence of a C-Reactive Protein (CRP) being ordered on the patient during the hospitalization before they were deemed to require transfer to the ICU;
- Age of the patient;
- Prior hospitalizations before the index hospitalization where there was a transfer to the ICU.

Our objective was to create an alert notification system for physicians. When the model's outcome surpasses the predetermined threshold, it will alert the doctor of increased risk of deterioration in the patient's health status. This will assist the physicians in prioritizing patients who may require additional attention. Such an alert would prompt the physician to act in order to prevent further deterioration. For example:

- Increase use of imaging in patients with infection with no confirmed source;
- Increase use of broad-spectrum antibiotics and/or infectious diseases specialist consultation in patients with signs of systemic infections;
- Increase use of advanced care settings such as Progressive Care Units (PCU) or Step-down units in order to monitor the patient better and avoid the need of the ICU admission;
- Consider other advanced procedures such as bronchoscopy, transesophageal echocardiograms, or incision/debridement interventions.

It would also help prevent certain heuristic errors in clinical decision-making, such as anchoring bias (Croskerry, 2003).

## 2. Materials and methods

### 2.1. Dataset

Records of patients who were transferred from non-intensive care units to intensive care units were queried from the Veteran Affairs Corporate Data Warehouse (CDW). We extracted all the admissions that resulted in ICU transfer for the period from 10/1/18 to 5/28/22. Two thousand four hundred twenty-five (2,425) such records were identified. The dataset did not contain any patient-identifiable information. The patient outcome was designated as a dependent variable, with bad outcome defined as the patient dying within 30 days of admission and good outcome as the patient being alive within 30 days of admission. For a complete list and description of variables, see Table 1.

**Table 1:** Description of variables.

**Variable**	**Description**
Outcome	0 = Good, 1 = Bad (Death within 30 days of admission)
Age	Age of the patient at the time of admission
ED visit flag	Was the patient seen first in the Emergency Room
Admit specialty	Admitting specialty name
Transfer ward	Intensive Care Unit (ICU) Type
Diagnosis MDC	Diagnostic Category
Infection MDC	Presence of infection
Platelet 25 drop flag	25% platelet drop
Lymphocytes 50 drop flag	50% lymphocytes drop
Platelet labs found	Were platelet results present
Platelet first lab value	Platelet values at admission
Platelet next lab value	Platelet values right after transfer to ICU
CRP labs found	Were CRP results present
Albumin labs found	Were Albumin results present
Lymphocytes first lab value	Lymphocytes values at admission
Lymphocytes next lab value	Lymphocytes values right after transfer to ICU
Hemoglobin labs found	Were hemoglobin results present
Hemoglobin first lab value	Hemoglobin results at admission
Hemoglobin next lab value	Hemoglobin results right after transfer to ICU
Admissions within Prev Year	Number of admissions in 12 months prior to the current admission
Admit to ICU transfer days	Days of stay from admission to ICU transfer
Total LOS	Total length of stay in the hospital

The veteran population is predominantly male, which can cause the ML models not to generalize well to female veterans (Cao et al., 2022). Unfortunately, due to the limited dataset (less than twenty-five hundred data points), we were not able to address this potential problem in the pilot study. We do plan to address it in a follow-up project with a vastly enlarged dataset.

The study has been approved by the Research and Development Committee of the James A. Haley Veterans' Hospital, which includes the Ethics Committee. They have classified it as a non-research Quality Improvement project.

### 2.2. Data cleaning and preparation

Columns with more than 20% missing values were deleted. Other missing values were replaced with “other” for categorical variables and with the mean value of the column for continuous variables. In addition, some numeric features were converted to categorical features based on histogram distribution, with the final dataset of 8 numeric features and 14 categorical features. Finally, the data was split into training/validation (90%) and testing (10%) using the Scikit-Learn Python library.

### 2.3. Data visualization

The AutoViz Python library was used to graph the dependent variable (outcome) relationship to various independent variables.

### 2.4. Model training and evaluation

**Figure 1:**
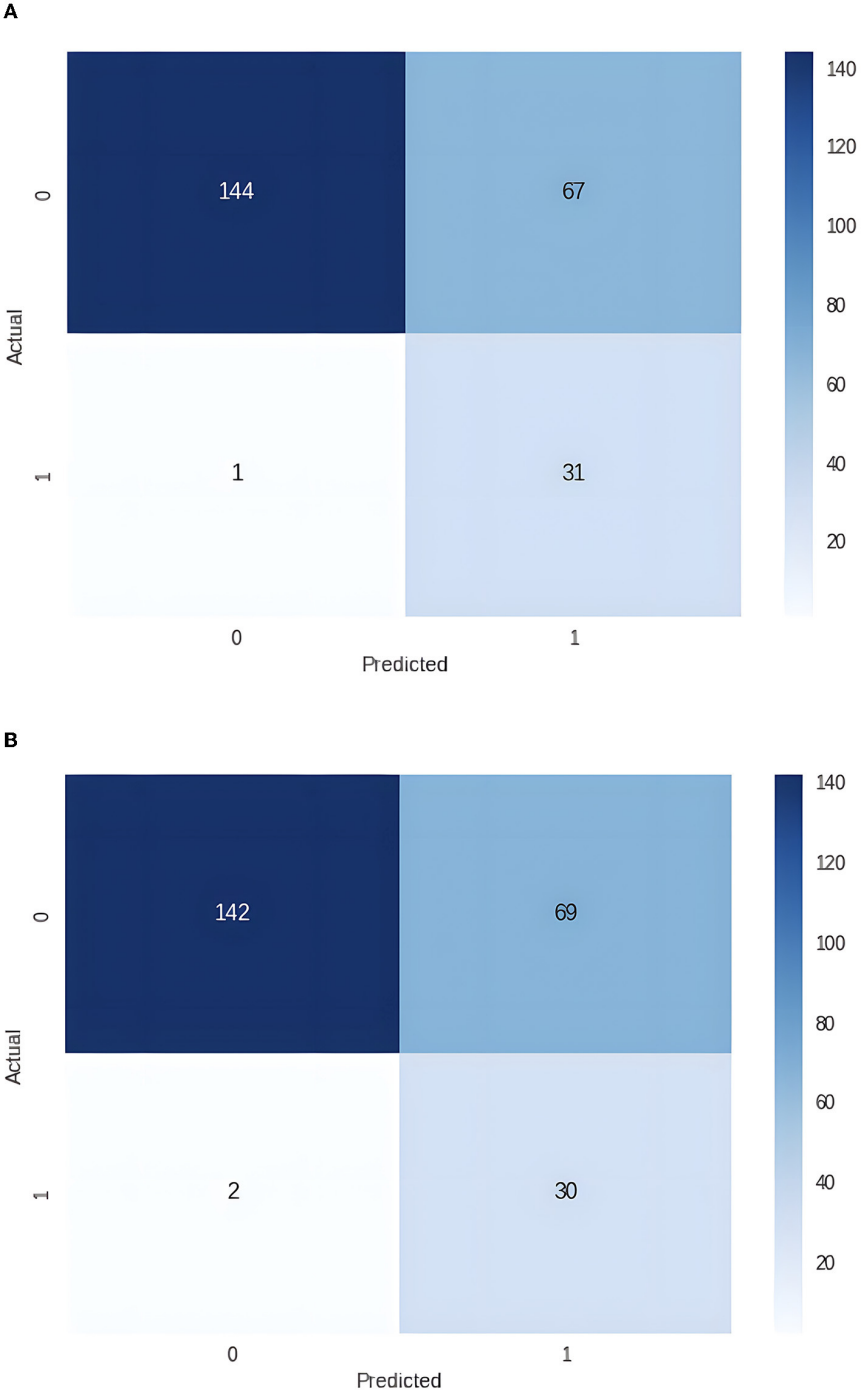
**(A)** Confusion matrix for the dataset with 22 independent variables (0 – good outcome, 1 – bad outcome). **(B)** Confusion matrix for the dataset with 20 independent variables (0 – good outcome, 1 – bad outcome).

The PyCaret Python library was used to train, evaluate, and test sixteen various machine-learning models, including Dummy Classifier, Random Forrest Classifier, Gradient Boosting Classifier, CatBoost Classifier, Extreme Gradient Boosting, Light Gradient Boosting Machine, Extra Tree Classifier, Ada Boost Classifier, Decision Tree Classifier, Logistic Regression, Ridge Classifier, Linear Discriminant Analysis, Naive Bayes, K Neighbor Classifier, Support Vector Machine (Linear Kernel), Quadratic Discriminant Analysis. Due to an imbalanced dataset (13% bad outcome, 87% good outcome), SMOTE (Synthetic Minority Oversampling Technique) was used to balance the training dataset. The six best-performing models were fine-tuned and evaluated on the unseen data from the testing dataset. In addition, we used 10-fold cross-validation for the training and fine-tuning of the top-performing models. The threshold was optimized for the best sensitivity to detect a bad outcome. The Light Gradient Boosting Machine (LightGBM) model showed the best results (see Figure 1A; Table 2A).

**Table 2A:** Results for the dataset with 22 independent variables.

**ML method**	**lr**	**GBC**	**LightGBM**	**qda**	**rf**	**xgboost**
Test accuracy	0.41	0.88	0.72	0.76	0.58	0.78
Test AUC	0.87	0.87	0.89	0.82	0.84	0.89
Test sensitivity	0.97	0.31	0.97	0.87	0.94	0.91
Test specificity	0.33	0.97	0.68	0.74	0.52	0.76
Threshold	0.1	0.5	0.1	0.4	0.2	0.1

lr, Logistic Regression; gbc, Gradient Boosting Classifier; LightGBM, Light Gradient Boosting Machine; qda, Quadratic Discriminant Analysis; rf, Random Forrest; xgboost, Extreme Gradient Boosting.

Since two independent variables, Length of Stay and Days to Intensive Care Unit Transfer, are unknown at the time of admission, we removed them from the dataset and repeated the experiments. Again, the best performance was achieved with the LightGBM (see Figure 1B; Table 2B).

**Table 2B:** Results for the dataset with 20 independent variables (without Total Length of Stay and Days to Intensive Unit Transfer).

**ML method**	**lr**	**GBC**	**LightGBM**	**qda**	**rf**	**xgboost**
Test accuracy	0.37	0.86	0.71	0.68	0.62	0.81
Test AUC	0.83	0.84	0.86	0.86	0.83	0.83
Test sensitivity	1	0.22	0.94	0.91	0.94	0.41
Test specificity	0.28	0.96	0.67	0.65	0.57	0.87
Threshold	0.1	0.5	0.3	0.4	0.2	0.5

lr, Logistic Regression; gbc, Gradient Boosting Classifier; LightGBM, Light Gradient Boosting Machine; qda, Quadratic Discriminant Analysis; rf, Random Forrest; xgboost, Extreme Gradient Boosting.

## 3. Results

The best results were obtained with the LightGBM models with both datasets, one that included Length of Stay and Days to Intensive Care Unit Transfer variables and the other without these two variables. The former achieved Receiver Operating Characteristics Curve-Area Under the Curve (ROC-AUC) of 0.89, an accuracy of 0.72, a sensitivity of 0.97, and a specificity of 0.68, while the latter achieved a ROC-AUC of 0.86, an accuracy of 0.71, sensitivity of 0.94 and specificity of 0.67, on the unseen testing dataset, see Table 2. The top five features for the former model were Total Length of Stay, Days to Intensive Care Transfer, Lymphocytes Next Lab Value, Lymphocytes First Lab Value, and Platelet First Value. For the latter model, the top five features were Lymphocytes First Lab Value, Hemoglobin First Lab Value, Hemoglobin Next Lab Value, Platelet First Lab Value, and Lymphocytes Next Lab Value (see Table 3).

**Table 3:** List of important features for the LightGBM (Light Gradient Boosting Machine) models.

**22 Features LightGBM model**	**20 Features LightGBM model**
Total Length of Stay	Lymphocyte First Lab Value
Admit to ICU Transfer Days	Hemoglobin First Lab Value
Lymphocyte Next Lab Value	Hemoglobin Next Lab Value
Lymphocyte First Lab Value	Platelet First Lab Value
Platelet First Lab Value	Platelet Next Lab Value

## 4. Discussion

Previous models looking at poor outcomes were mostly based on length of stay and days to transfer to the ICU (Paoli et al., 2018). Such models do not help physicians proactively manage patients and recognize possibility of clinical deterioration early enough to prevent ICU transfer. Our artificial intelligence project was designed to do just that. It clearly shows that there are other factors (change in lymphocyte count values, hemoglobin values, or platelet count values) that can be an early warning sign of deterioration. AI can be invaluable in identifying these factors in a timely manner.

We can explore numerous future directions regarding this project's expansion. One direction would be to ensure that the hemoglobin drop and platelet count drop are not occurring in the setting of overt disseminated intravascular coagulation (DIC). Our project added the lymphocyte count drop, which is not part of the DIC process and related very consistently with the hemoglobin drop and the platelet count drop. The lymphocyte count, in particular, had become an essential acute phase reactant item in the face of the COVID-19 pandemic, where such dramatic lymphocyte count drops were noted when the patient started to become dramatically ill (Illg et al., 2021).

The future direction of this project is to continue to build on additional factors that refine the predictability model of worsening of the patient's condition once admitted to a general medical or surgical floor. For example, future projects could look at the relationship between vital signs changes in heart rate and systolic blood pressure readings, especially if they remain in what would be considered normal range but still changed from the patient's baseline values during the initial parts of the hospitalization. In addition, future studies may also include variables like temperature, blood pressure, ventilation, ICD9/10 codes, and vasopressor data, among others.

Developing an alert for physicians to help them identify patients objectively from their laboratory values such as changes in hemoglobin, platelet count, and lymphocyte count would be a beneficial for medical and surgical house officers who take care of numerous patients on shift work and often do not perform a direct evaluation of the patient at the beginning of their shifts or even during the duration of their shifts. Therefore, implementing an alert system would aid in identifying patients who need extra attention during the shift and prioritizing their care.

As hospitals develop teams of infectious disease physicians and intensivists to help create high-reliability organizational states, a model like the one we have brought forward in this paper could push required targeted reviews of patients by such infectious disease physicians and intensivists. This would include such interventions as (a) vital signs being run at a much higher frequency than every shift, (b) vital orthostatic signs being obtained on such at-risk patients, (c) employing therapeutic drug monitoring on patients receiving antibiotic therapy to ensure that drug levels are reaching expected levels, (d) consideration for expedited imaging orders depending on the patient clinical situation, and (e) consideration in academic hospital models of review of patients by attending physicians if the medical house officer is a resident in training.

Our pilot study created the AI predictive mortality model prototype that assists providers and frontline staff in managing large patient loads during shift change by helping them focus their efforts toward improving patient outcomes. The key limitation of our study is the small data size. Undersized data can lead to bias by underrepresenting certain groups, for example, women and minorities (Gianfrancesco et al., 2018). In addition, including more clinically relevant variables could be helpful. Further studies with more variables and a larger, diverse, and randomized dataset are needed to make such a model more robust and to generalize better to various patient populations.

## Data availability statement

The datasets presented in this article are not readily available because they belong to Veterans Affairs and cannot be shared. Queries should be directed to the corresponding author.

## Author contributions

JL, GA, CJ, AK, MK, and AB: study conception and design, data collection, analysis and interpretation of results, and manuscript preparation. All authors contributed to the article and approved the submitted version.
